# The sweet potato B-box transcription factor gene *IbBBX28* negatively regulates drought tolerance in transgenic Arabidopsis

**DOI:** 10.3389/fgene.2022.1077958

**Published:** 2022-11-29

**Authors:** Jingjing Dong, Cailiang Zhao, Jie Zhang, Yuchao Ren, Liheng He, Ruimin Tang, Wenbin Wang, Xiaoyun Jia

**Affiliations:** ^1^ College of Agriculture, Shanxi Agricultural University, Taigu, Shanxi, China; ^2^ College of Life Sciences, Shanxi Agricultural University, Taigu, Shanxi, China

**Keywords:** sweet potato, *IbBBX28*, Arabidopsis, drought tolerance, yeast two-hybrid

## Abstract

B-box (BBX) which are a class of zinc finger transcription factors, play an important role in regulating of photoperiod, photomorphogenesis, and biotic and abiotic stresses in plants. However, there are few studies on the involvement of BBX transcription factors in response to abiotic stresses in sweet potato. In this paper, we cloned the DNA and promoter sequences of *IbBBX28*. There was one B-box conserved domain in IbBBX28, and the expression of *IbBBX28* was induced under drought stress. Under drought stress, compared to wild type Arabidopsis, the protective enzyme activities (SOD, POD, and CAT) were all decreased in *IbBBX28*-overexpression Arabidopsis but increased in the mutant line *bbx28*, while the MDA content was increased in the *IbBBX28*-overexpression Arabidopsis and decreased in the *bbx28*. Moreover, the expression levels of the resistance-related genes showed the same trend as the protective enzyme activities. These results showed that *IbBBX28* negatively regulates drought tolerance in transgenic Arabidopsis. Additionally, the yeast two-hybrid and BiFC assays verified that IbBBX28 interacted with IbHOX11 and IbZMAT2. The above results provide important clues for further studies on the role of *IbBBX28* in regulating the stress response in sweet potato.

## Introduction

In nature, plants are constantly stressed by adverse abiotic environmental factors such as drought, heat, cold, nutrient deficiencies and excess salt or toxic metals in the soil. Moreover, abiotic stresses such as high salt and drought are environmental factors that affect crop yield and quality (Munns and Tester, 2008; Zhai et al., 2016; Bailey-Serres et al., 2019). Revealing the mechanisms of plant response to abiotic stress will benefit the development of resistant crops and improve agricultural sustainability. Plants will produce a series of physiological and biochemical reactions by drought stress, including stomatal closure, reduced photosynthesis and cell growth. When plants are under drought stress, the hormone ABA is also produced and the expression of stress-related genes are induced. Many transcription factors play a role in regulating drought stress signal transduction pathways, such as ARED, MYC, MYB, bZIP, NAC and BBX (Shinozaki and Yamaguchi-Shinozaki, 2007; Yang et al., 2014).

Zinc finger structure transcription factors are one of the important families in plants, and B-box (BBX) is one subgroup of zinc finger proteins. BBX transcription factor is of great interest due to its many functions in plant growth and development (Khanna et al., 2009; Crocco and Botto, 2013; Gangappa and Botto, 2014; Song et al., 2020). *STO* (salt tolerace)/*AtBBX24* gene responses to salt stress and promotes the root growth of Arabidopsis under high salt conditions (Nagaoka and Takano, 2003). It was found that the chlorophyll content and net photosynthetic rate were lower in the overexpressed *SlBBX17* tomato, and the heat tolerance of the transgenic tomato increased (Xu et al., 2022). *CmBBX24* (homologous to *AtBBX24*) which negatively regulated the expression of genes in photoperiodic flowering pathway including *GI*, *PRR5*, *CO*, *FT*, and *SOC1*, delayed the flowering time in chrysanthemum. In addition, *CmBBX24* is involved in regulating the response of chrysanthemum to low temperature and drought by regulating the stress response and GA synthesis-related genes (Yang et al., 2014). Overexpression of *IbBBX24* and *IbPRX17* in sweet potato, the salt and drought tolerance were significantly improved, and the molecular mechanisms of the IbBBX24-IbTOE3-IbPRX17 model in response to abiotic stresses were resolved (Zhang et al., 2022). BBX family members also play important roles in hormone signal transduction pathways. Studies have found that Arabidopsis *BBX* gene responds to plant hormones and is involved in various hormone pathways (Vaishak et al., 2019). When dealing with exogenous hormones (ABA, GA, JA and SA), the transcription levels of several *BBX* genes were increased. The promoters of these *BBX* genes contain one or more hormone-responsive *cis*-acting elements, such as ABRE (ABA response element), ERE (ethylene response element), CGTCA motifs, and TGACG (MeJA response element) (Gangappa and Botto, 2014; Chu et al., 2016).

The sweet potato [*Ipomoea batatas* (L.) Lam.] is an important root vegetable, and rank the seventh largest food crop in the world. Compared with other crops, sweet potato has many advantages, such as high adaptability, rapid growth, and high yield (O'BRIEN, 1972; Bovell-Benjamin, 2007; Kwak, 2019). The tuberous roots of sweet potato contain high levels of nutrients, such as starch, protein, fatty acids, sugars, and vitamins, and are also rich in inorganic salts such as calcium, phosphorus, and iron, as well as carotenoids and anthocyanins. It plays a vital role in human health for its antioxidant, anticancer, anti-aging, and immunomodulatory effects, and its nutritional value is gradually being recognized (Abegunde et al., 2013; Kwak, 2019; Nguyen et al., 2021).

Several genes related to abiotic stress tolerance were isolated and identified from sweet potato and have been used to improve abiotic stress resistance of sweet potato (Liu, 2017; Lyu et al., 2021). In Arabidopsis, LOW OSMOTIC STRESS 5 (LOS5)/ABA3 is an important regulator in response to cold, salt, and drought stresses, and overexpression of *AtLOS5* in sweet potato improves its salt tolerance (Gao et al., 2011). The sweet potato bZIP transcription factor gene *IbbZIP1* improved salt and drought tolerance in Arabidopsis (Kang et al., 2019). With the overexpression of *IbP5CR* gene in sweet potato, the ROS scavenging system of the transgenic sweet potato was activated, and the salt resistance was improved (Liu et al., 2014). Co-expression of the Arabidopsis Na^+^/H^+^ reverse transporter NHX1 and the DEAD-box RNA decapping enzyme elF4A1 in sweet potato enhanced its drought tolerance (Zhang et al., 2019).

It was found that *AtBBX28* negatively regulates photomorphogenesis (Lin et al., 2018), and *AtBBX28* negatively regulates flowering in Arabidopsis by co-acting with *CO* and *FT* (Liu et al., 2020). However, there are few studies on the response of *IbBBX28* to stress in sweet potato. In this paper, we conducted a preliminary study on the biological function of *IbBBX28*. The *IbBBX28*-overexpression Arabidopsis was obtained, and the regulatory role of *IbBBX28* in Arabidopsis drought stress response was explored. Furthermore, the yeast two-hybrid assay and bimolecular fluorescence assay (BiFC) were used to screen and validate the interacting proteins of IbBBX28. The mechanism of *IbBBX28* in the regulation of stress response in sweet potato was initially explored.

## Materials and methods

### Plant materials and growth conditions

The sweet potato variety Xuzishu-3 (XZ-3) was planted in the experimental field in Shanxi Agricultural University. *Nicotiana benthamiana* was used for the BiFC assay of the interaction between IbBBX28 and its interacting protein. The growth condition of *N. benthamiana* was as follows: light/dark for 16/8 h at 26°C. The seeds of *Arabidopsis thaliana* (Columbia) were kept by our laboratory. The *bbx28* T-DNA insertion *Arabidopsis thaliana* (SALK_094193C) was obtained from AraShare Technology Service Center (https://www.arashare.cn) and analyzed by PCR. The growth condition of *Arabidopsis thaliana* was as follows: light/dark for 16/8 h at 22°C.

### Cloning and sequence analysis of *IbBBX28* and its promoter

The Open Reading Frameb (ORF) sequence of *IbBBX28* (GenBank accession number OP047916) was previously cloned in our lab, and the gene structure of the genomic DNA sequence of *IbBBX28* was predicted from the database (https://121.36.193.159/blast.html). The genomic DNA was extracted from XZ-3 root sample using the CTAB method (Safeena et al., 2021). Using the genomic DNA as template, the genomic DNA sequence and promoter sequence of *IbBBX28* were cloned by PCR. All the specific primers are listed in Supplementary Table S1. Based on the DNA and ORF sequences of *IbBBX28*, the gene structure was mapped using the online tool Gene Structure Display Server (http://gsds.gao-lab.org/). Multiple protein sequence alignments of BBX28 from different species were conducted with DNAMAN software. The conserved domain was confirmed by the Conserved Domain Database (CDD) (https://www.ncbi.nlm.nih.gov/Structure/cdd/wrpsb.cgi). A maximum-likelihood (ML, Maximum-likelihood) phylogenetic tree of the BBX proteins was constructed using MEGA 7.0 software. The bootstrap was set at 1,000 replicates (Kumar et al., 2016). The *cis*-acting elements of the *IbBBX28* promoter were predicted at the PlantCARE website (http://bioinformatics.psb.ugent.be/webtools/plantcare/html/) (Lescot et al., 2002).

### Expression analysis of *IbBBX28* in sweet potato

Approximately 10 cm vines were cut from XZ-3 plants grown in the field for 30 d and placed in 1/2 Hoagland nutrient solution for hydroponics in an incubator (25°C/16 h of light and 22°C/8 h of darkness). After 2 weeks of growth, 30% PEG6000 was added to the 1/2 Hoagland nutrient solution for drought stress treatments. Samples were taken at 0, 6, 12, 24, 48, and 72 h after the stress treatment. All treatments had three biological replications. The samples were frozen in liquid nitrogen and kept in a −80°C refrigerator for further use.

Total RNA was extracted using a Quick RNA Isolation kit (Huayueyang Biotech, Beijing, China). Using 1 μg total RNA as template, a PrimeScript RT Reagent kit with gDNA Eraser (Takara Bio, Shiga, Japan) was used to synthesis cDNA by following the manufacturer’s instructions.

RT-qPCR was used to analyze the expression pattern of the *IbBBX28* gene in XZ-3 leaves, stems, and tubers under different PEG treatment times (0, 6, 12, 24, 48, and 72 h). The experiments were conducted on a CFX96PCR system (Bio-Rad, United States), and the reaction system was 10 μL (1 μl cDNA, 5 μl SYBR®Premix Ex-TaqTM, 3.2 μL dd H_2_O, 0.4 μL PF and 0.4 μl PR). All the experiments were replicated three times, the relative expression was calculated using the 2^−ΔΔCt^ method, and the *IbActin* gene (AY905538) was used as an internal reference (Gao et al., 2011).

### Drought tolerance analysis

The overexpressed vector pCAMBIA1300-*IbBBX28* (pC1300-*IbBBX28*) was constructed and the positive transgenic plants were obtained. The seeds of wild type Arabidopsis, T_3_ generation of two *IbBBX28*-overexpression Arabidopsis lines (OE-4 and OE-5), and mutant line *bbx28* were sown on 1/2 MS solid medium (Petri dishes of 90 mm diameter), respectively. After 7 d of growth on plates, the seedlings were transferred to 1/2 MS solid medium (150 mm diameter dishes) containing different concentrations (0, 200, 250, and 300 mM) of mannitol. Samples were taken after 7 d of treatment.

Seedlings (WT, OE-4, OE-5, and *bbx28*) which were grown on 1/2 MS solid medium for 7 d, were transplanted to pots (1:3 ratio of nutrient soil to vermiculite). After 15 d of normal growth in the incubator, the plants were rehydrated for 3 d after 10 d of drought treatment. Samples were taken after 7 d of treatment. The plant materials collected were immediately frozen in liquid nitrogen and stored in a −80°C freezer for further use. The protective enzyme activities (CAT, POD, and SOD) and MDA content were determined according to the kit instructions (Nanjing Jiancheng Biotechnology Company, Nanjing, China).

### Expression analysis of related genes

RNA from the leaves of the WT, T_3_ generation Arabidopsis of overexpressed *IbBBX28*, and *bbx28* grown normally and treated with drought stress for 7 d was extracted and then reverse transcribed to cDNA, and the expression levels of the related resistance genes were analyzed using RT-qPCR. All the primers are shown in Supplementary Table S2. All the experiments were repeated three times, and the relative expression levels were calculated in the 2^−ΔΔCt^ method. The Arabidopsis *Actin* gene (NM_112764) was used as the internal control (Kang et al., 2018).

### Yeast two-hybrid assay

The yeast two-hybrid (Y2H) assay was carried on via co-transformation method according to the protocols (OE biotech. Company). The vectors of pGADT7 and pGBKT7 were used as the prey and the bait constructs, respectively. The cDNA from sweet potato tuberous root was constructed into the prey vector pGADT7 to obtain yeast library. The coding sequence (CDS) of *IbBBX28* was inserted into the empty vector pGBKT7 with *Eco*RI and *Bam*HI sites to construct the bait vector pGBKT7-IbBBX28. The CDS of *IbHOX11* and *IbZMAT2* were insert into the empty vector pGADT7 with *Eco*RI and *Bam*HI sites to obatin the prey vectors of pGADT7-IbHOX11 and pGADT7-IbZMAT2. The bait and prey vectors were co-transformed into yeast strain Y2H Gold, and positive clones were screened. The transformed yeast broth was screened on a screening plate SD/-Trp/-Leu/-His/-Ade/X-α-Gal/AbA and incubated in an incubator at 30°C for 3–5 d in an inverted position.

### BiFC assay

The CDS sequence of *IbBBX28* was constructed into the pSm35s-nYFP vector and named NY-IBBBX28. The CDS sequences of the interacting protein coding genes were constructed into the pSm35s-cYFP vector and named IbHOX11-CY and IbZMAT2-CY, respectively. The primers required for the BiFC assay are shown in Supplementary Table S3. *Bam*HⅠ and *Xba*Ⅰ were selected as the restriction sites, and the constructed vector plasmid was transformed into *Agrobacterium* GV3101 for the BiFC assay. *Agrobacterium* tumefaciens was inoculated into 10 ml LB liquid medium with spectinomycin and incubated in a shaker (200 rpm) at 28°C for 1 h. Then, it was centrifuged at 4,000 rpm for 10 min, the supernatant was discarded, and the bacterial cell was resuspended (10 mM MgCl_2_ and 120 μM AS). The OD_600_ was adjusted to about 0.6. The *Agrobacterium* solutions of the two genes were mixed in a ratio of 1:1, and left at room temperature for 3 h, and then injected with four to five leaf stage tobacco leaves. The injected tobacco was incubated at 26°C (16 h light/8 h dark) for 2 days; then, it was observed and photographed with a laser scanning confocal microscope (Nikon C2-ER). The excitation wavelength of yellow fluorescent protein YFP was 511 nm, and the emission wavelength was 525 nm.

### Statistical analysis

Three biological replicates were performed for all experiments, and the data are presented as the mean value ±standard deviation (SD). All data were analyzed with SPSS software (Chicago, IL, United States, version 8.0) using *t*-test or one-way ANOVA and least significant difference (LSD) test.

## Results

### Sequence analysis of *IbBBX28* and its promoter

**FIGURE 1:**
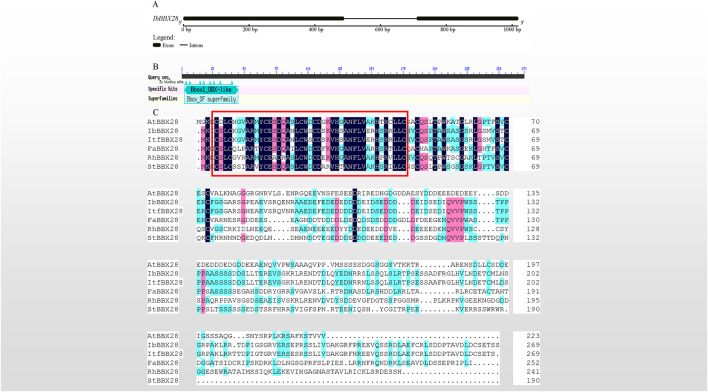
Sequence cloning of *IbBBX28*, gene structure, and multiple sequence alignment results. **(A)** The gene structure of *IbBBX28*. **(B)** The structural domain of IbBBX28 obtained by the CD-search in the NCBI. **(C)** The multiple sequence alignment of IbBBX28 and BBX28 proteins in other species; the sequence in the red box is the B-box domain. The amino acids sequences are: AtBBX28 (*Arabidopsis thaliana*, AT4G27310), ItfBBX28 (*I. trifida*;Itr_sc000359.1_g00035.1), FaBBX28 (*Fragaria* x *ananassa*, QOI16737), RhBBX28 (*Rosa hybrid cultivar*, UCU84643), and StBBX28 (*Solanum tuberosum*, ARU77867).

In this study, a 1,022 bp genomic sequence of *IbBBX28* was obtained with two exons and one intron (Figure 1A). The ORF encoding protein of IbBBX28 contained one BBX transcription factor specific domain B-box in the 3–45 region of its amino acid sequence (Figure 1B). Multiple sequence alignment of IbBBX28 and BBX28 proteins from other species showed that they all contain one B-box domain (Figure 1C). The IbBBX28 protein sequence has the highest similarity with *Ipomoea trifida* (Itr_sc000359.1_g00035.1) at 95.57%. The similarities of IbBBX28 with *Arabidopsis thaliana* (AT4G27310), *Fragaria* x *ananassa* (QOI16737), *Rosa hybrid cultivar* (UCU84643), and *Solanum tuberosum* (ARU77867) were 29.89%, 40.21%, 31.48%, and 30.74%, respectively.

The phylogenetic tree of the above six species was constructed. It showed that IbBBX28 had the closest evolutionary relationship with ItfBBX28, a sweet potato diploid wild ancestor (Supplementary Figure S1).

The 2000 bp upstream sequence of ATG of *IbBBX28* was cloned and named *IbBBX28*-Pro. The *cis*-acting element analysis showed (Supplementary Table S4) that *IbBBX28*-Pro contain six stress-responsive elements (in response to anaerobic, low temperature, drought, and other stresses), 10 hormone-responsive elements (GA, abscisic acid, methyl jasmonate, and salicylic acid), 19 photoresponsive elements and 34 RNA polymerase binding sites TATA box.

### The expression of *IbBBX28* is induced under drought stress in sweet potato

**FIGURE 2:**
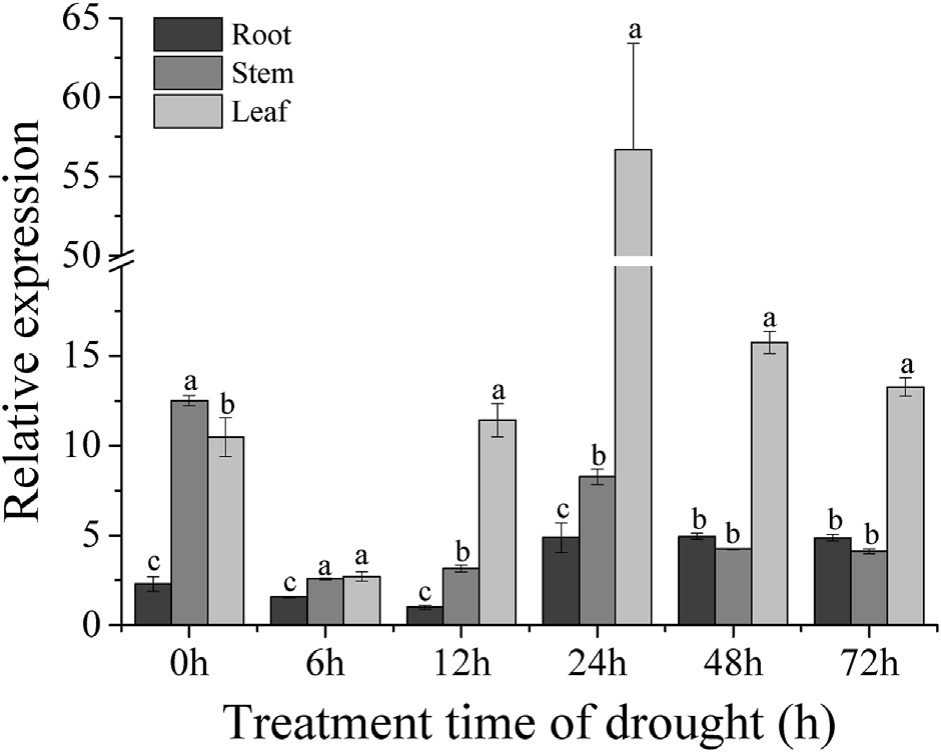
Expression analysis of *IbBBX28* under PEG stress treatment. The expression of *IbBBX28* at 12 h in root was set as 1-fold. Data are presented as means ± SD (*n* = 3). The different letters indicate significant differences in the expression level of the *IbBBX28* gene in the roots, stems, and leaves under the same time treatment (*p* < 0.05) by one-way ANOVA and least significant difference (LSD) test.

The sweet potato variety XZ-3 was subjected to drought stress with 30% PEG6000. Samples of the root, stem, and leaf were taken at 0, 6, 12, 24, 48, and 72 h after PEG treatment. RNA was extracted from root, stem, and leaf tissues, and these RNA were reverse transcribed into cDNA. The expression levels of the *IbBBX28* gene in the three tissues was analyzed. The results showed that the expression of the *IbBBX28* gene was significant induced (Figure 2) under PEG treatment. Compared with 0 h, the expression level of the *IbBBX28* gene in leaf and root were peaked at 24 h with 5.5 and 2 folds, respectively. And the expression of *IbBBX28* was decreased in stem. The results indicated that *IbBBX28* response to drought stress.

### Overexpression of *IbBBX28* negatively regulates drought tolerance in transgenic Arabidopsis

To study the function of the *IbBBX28* gene, the overexpression vector pC1300-*IbBBX28* was transformed into *Arabidopsis Thaliana*. Seven transgenic Arabidopsis lines were obtained, and two lines (OE-4 and OE-5) in which the expression level of *IbBBX28* gene was relatively high were chosen for further study.

**FIGURE 3:**
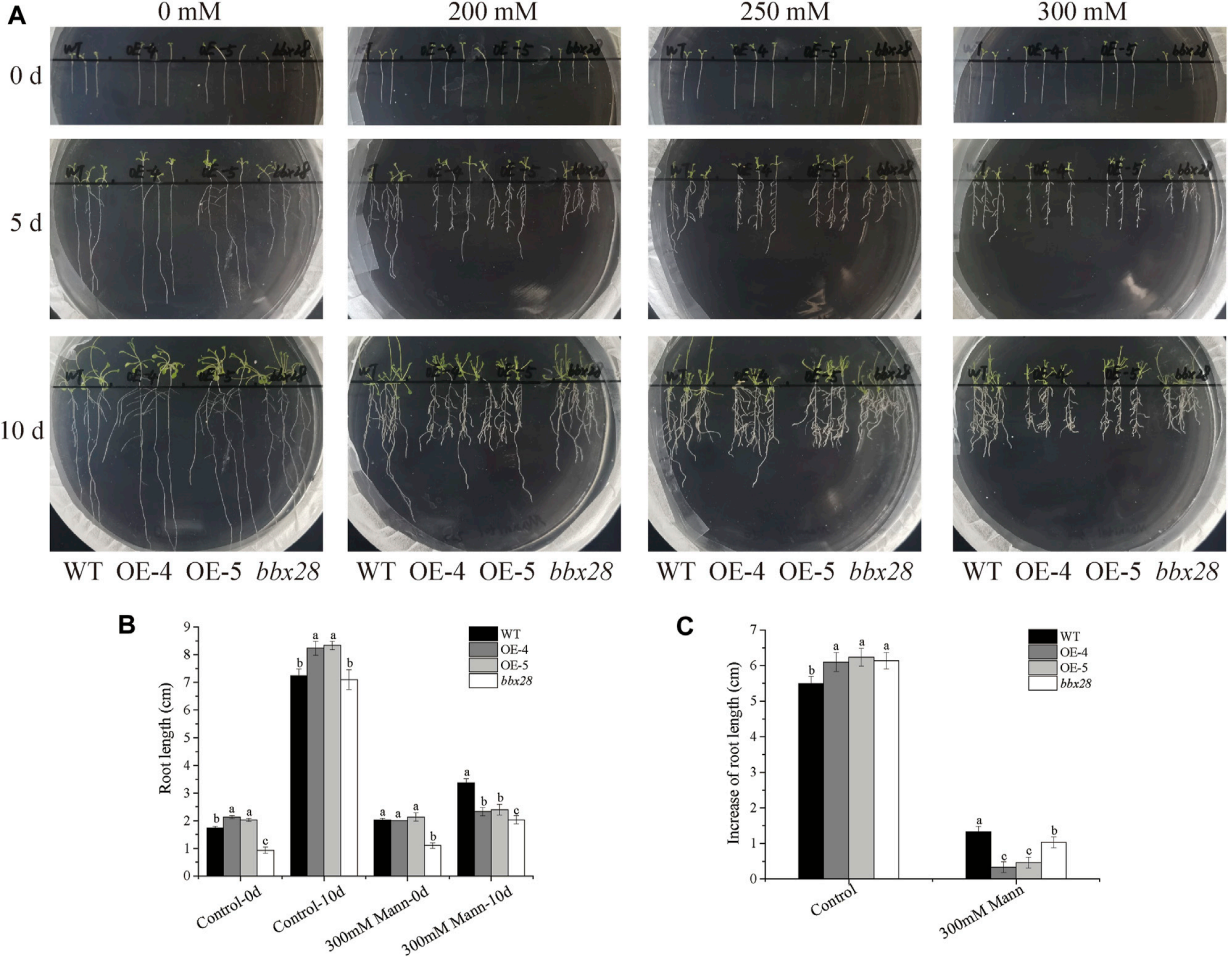
The phenotype of overexpressing *IbBBX28* transgenic Arabidopsis and *bbx28*T-DNA insertion Arabidopsis in 1/2MS medium supplemented with different concentrations of mannitol. **(A)** The growth of overexpressing *IbBBX28* transgenic Arabidopsis in 1/2 MS medium supplemented with 0, 200, 250, and 300 Mm mannitol. **(B)** The root length. **(C)** The root elongation. Data are presented as means ± SD (*n* = 3). Different lowercase letters indicate significant differences between OE-4, OE-5, *bbx28*, and WT (*p* < 0.05) by one-way ANOVA and least significant difference (LSD) test.

A mannitol-simulated drought stress test was carried out on the wild type (WT), *IbBBX28*-overexpression Arabidopsis, and *bbx28* mutant plant (Figure 3). In the medium without mannitol addition, there was no significant difference in the growth of the four lines. After adding different concentrations of mannitol to the medium, the growth of the WT, OE-4, OE-5 and *bbx28* were inhibited, and the root growth was slow. Compared with the WT, the root growth of the OE-4, OE-5, and *bbx28* was less (Figure 3A). On the medium supplemented with 0 mM mannitol, the root length and root elongation of the OE-4 and OE-5 plants were slightly longer than the WT after 10 days of growth. The root length of the *bbx28* was not significantly different from the WT, and the root elongation was slightly larger than the WT (Figures 3B,C). On the medium supplemented with 300 mM mannitol, the root length and root elongation of the OE-4 and OE-5 plants were lower than those of the WT plants after 10 days of growth. The root length of the *bbx28* was lower than that of the WT, but the root elongation was not significantly different from that of the WT (Figures 3B,C).

**FIGURE 4:**
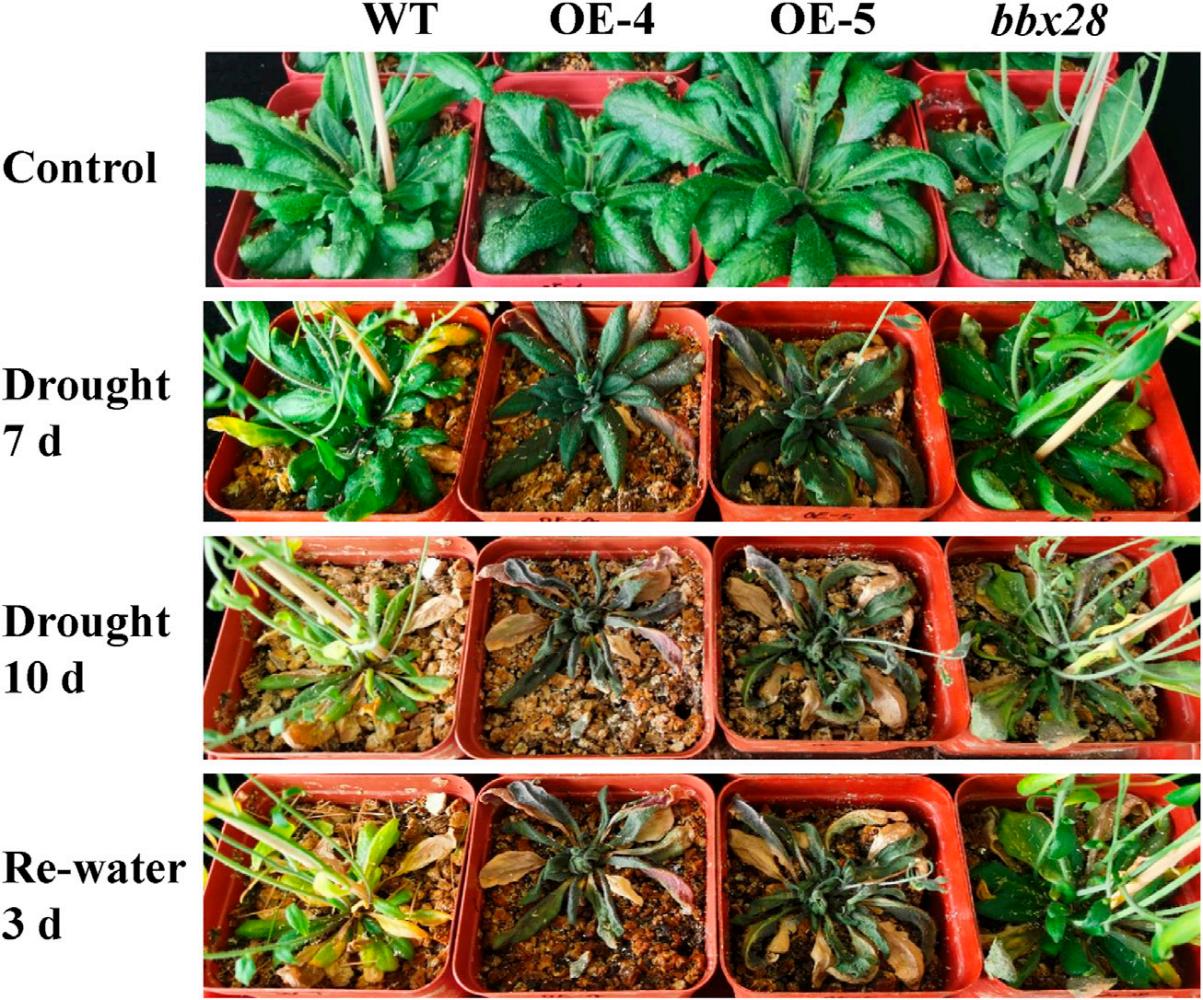
The phenotype of overexpressing *IbBBX28* transgenic Arabidopsis and *bbx28* T-DNA insertion Arabidopsis under drought stress. The experiments were performed three times with similar results. There were more than 10 plants in each replication. The photographs depict the results of one of the three experiments.

A soil drought stress test was conducted (Figure 4). The results showed that after 10 days of drought stress, the growth of OE-4 and OE-5 was significantly worse than that of the WT, while the growth of the mutant line *bbx28* was better than that of WT. After rehydration, the growth of the WT and *bbx28* recovered somewhat, however, the growth of OE-4 and OE-5 were still worse.

**FIGURE 5:**
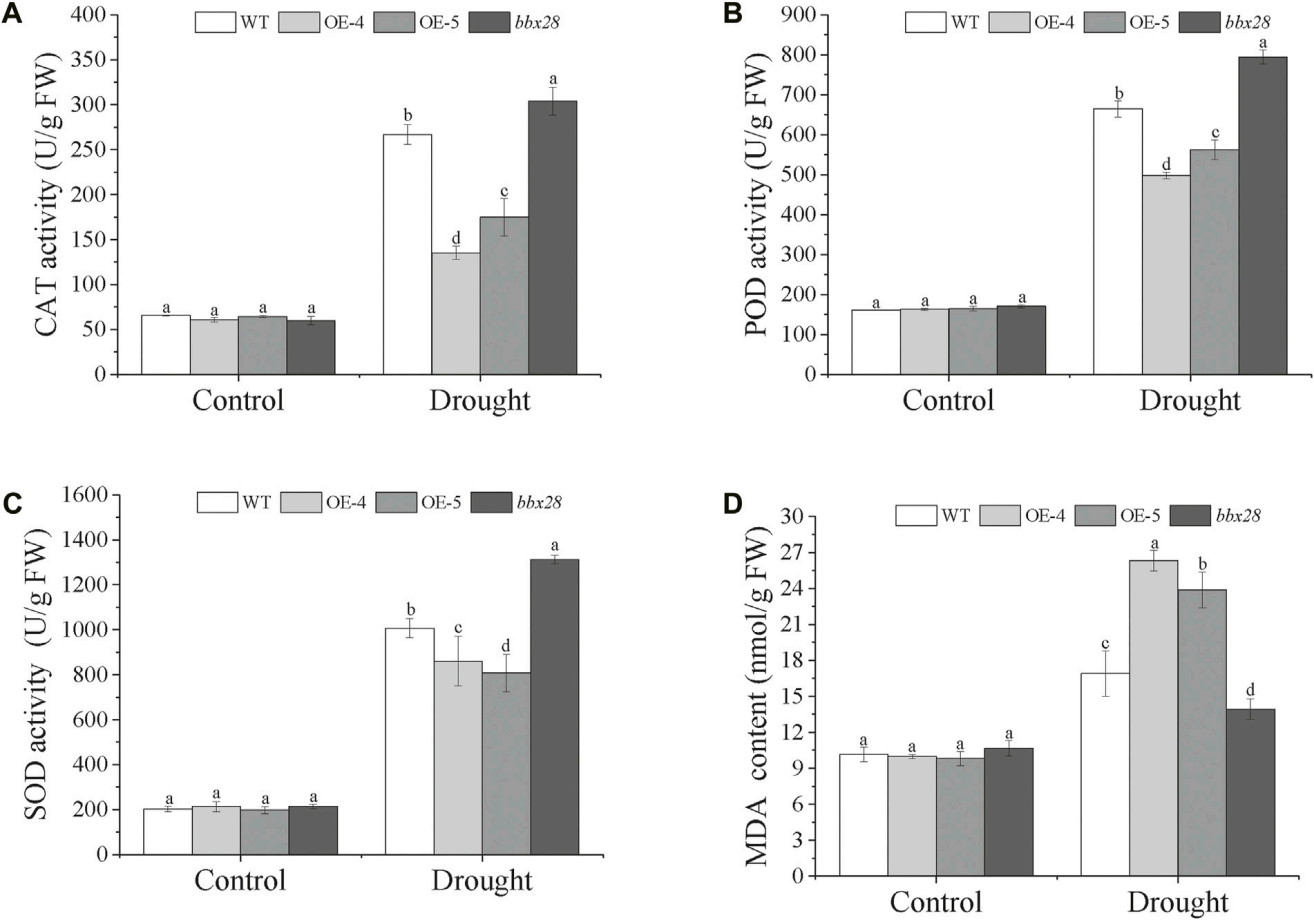
The activity of superoxide dismutase (SOD), peroxide (POD), and catalase (CAT), and the content of malondialdehyde (MDA) in the transgenic Arabidopsis and WT plants under drought stress. **(A)** The activity of CAT (U/g FW). **(B)** The activity of POD (U/g FW). **(C)** The activity of SOD (U/g FW). **(D)** The content of MDA. Data are presented as means ± SD (*n* = 3). The different letters indicate significant differences among OE-4, OE-5, *bbx28*, and WT at *p* < 0.05 by one-way ANOVA and least significant difference (LSD) test.

The activity of the protective enzymes was measured (Figures 5A–C). Under normal growth conditions (Control), the activities of superoxide dismutase (SOD), and peroxide (POD), and catalase (CAT) in the OE-4, OE-5, *bbx28*, and the WT showed no significant differences. Under drought stress, compared with the Control, the activities of the CAT, POD, and SOD in the WT, OE-4, OE-5, and *bbx28* were increased, but the enzyme activities in the WT were significantly higher than those in OE-4 and OE-5 and lower than those in *bbx28*. The results of the malondialdehyde (MDA) content determination (Figure 5D) showed that under normal growth conditions, there was no significant difference in MDA content among the WT, OE-4, OE-5, and *bbx28*. Under drought stress, the MDA content in WT, OE-4, OE-5, and *bbx28* was significantly increased compared with the Control, but the MDA content in the OE-4 and OE-5 was significantly higher than that in the WT, while the MDA content in the WT was higher than that in the *bbx28*. In conclusion, combined with the results of the drought stress tests in the Arabidopsis seedlings, it is speculated that *IbBBX28* negatively regulates the drought tolerance of Arabidopsis.

### Overexpression of *IbBBX28* down regulates the expression of the stress-responsive genes

**FIGURE 6:**
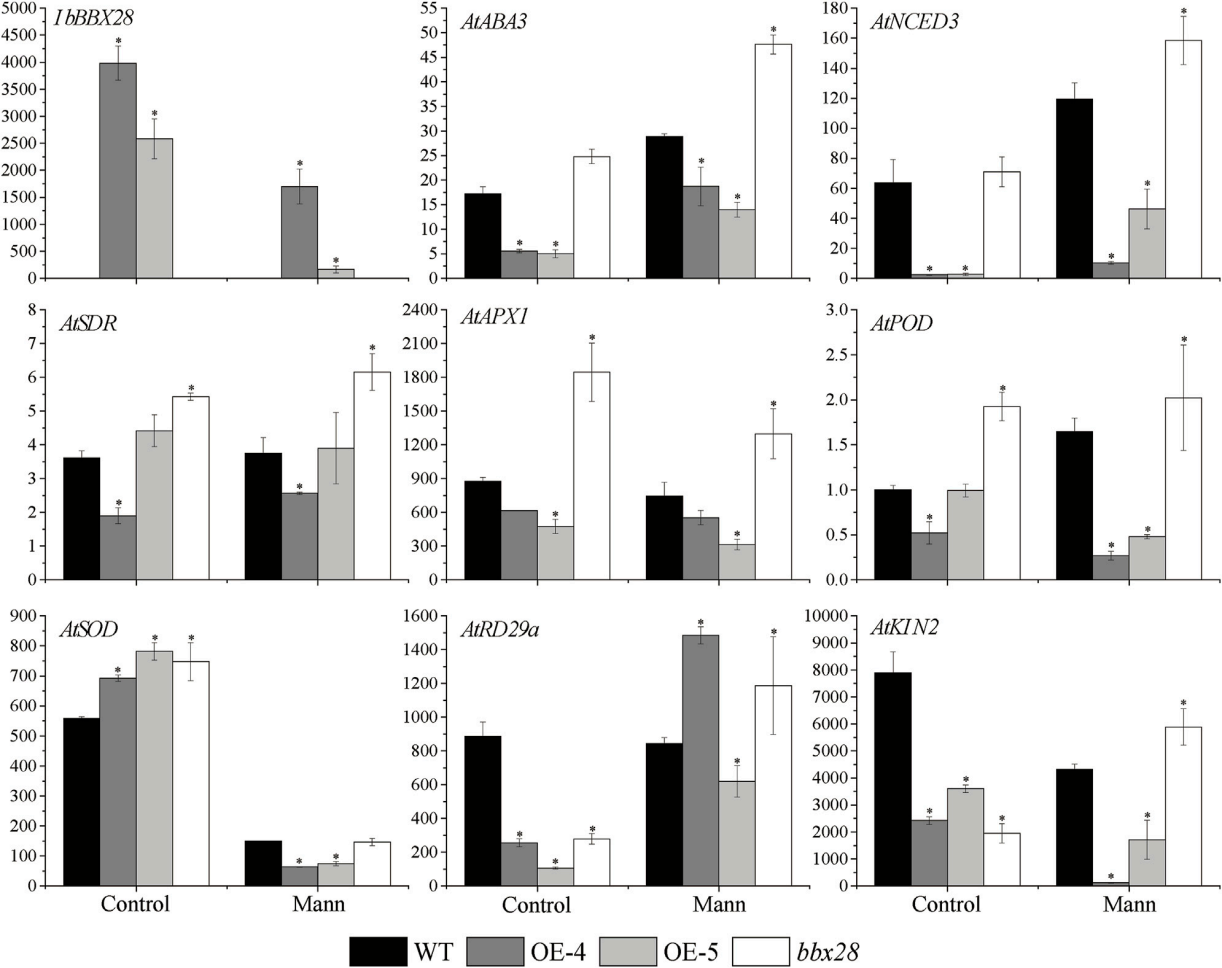
Expression analysis of overexpressing *IbBBX28* transgenic Arabidopsis resistance related genes under drought stress. The expression of *AtPOD* in WT in control group was set as 1-fold. Data are presented as means ± SD (*n* = 3). * indicates a significant difference of OE-4, OE-5, and *bbx28* from that of the WT at *p* < 0.05 by the *t*-test.

RNA was extracted from the WT, transgenic lines (OE-4, OE-5), and mutant lines (*bbx28*) under normal and drought treatment for 7 days. The expression of the resistance-related genes was analyzed by RT-qPCR after reverse transcription into cDNA (Figure 6). The results showed that the expression level of *IbBBX28* in the OE-4 and OE-5 decreased after drought stress compared with the control group. The expression levels of the *AtNCED3*, *AtABA3*, and *AtSDR* genes involved in ABA biosynthesis were increased in the four lines, but the expression levels in the WT were higher than those in the OE-4 and OE-5 and lower than those in the *bbx28*. The expression of the stress response gene *AtRD29a* was significantly increased, and the expression of the *AtRD29a* in the WT was higher than in the OE-4 and OE-5 and lower than in the *bbx28*. The expression of *AtKIN2* in the WT was decreased, but the expression of *AtKIN2* in the WT was higher than that in the OE-4 and OE-5 and lower than that in the *bbx28*. After drought stress treatment, the expressions of *AtAPX1*, *AtSOD* and *AtPOD* were decreased, but their expressions in the WT were higher than those in the OE-4 and OE-5 and lower than those in the *bbx28*.

### IbBBX28 interacts with IbHOX11 and IbZMAT2

**FIGURE 7:**
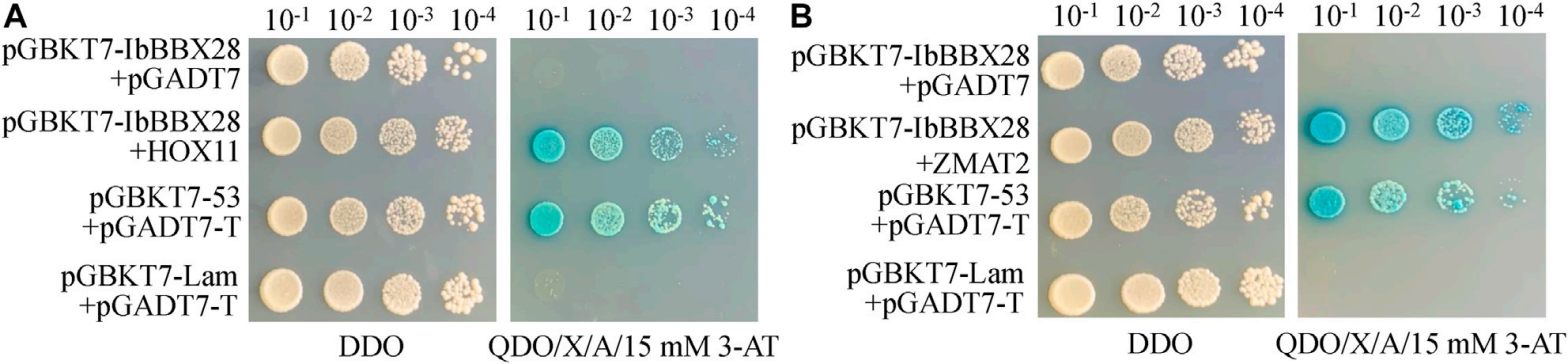
Plate validation of IbBBX28 and interacting proteins. **(A)** Plate validation of IbBBX28 and IbHOX11. **(B)** Plate validation of IbBBX28 and IbZMAT2.

**FIGURE 8:**
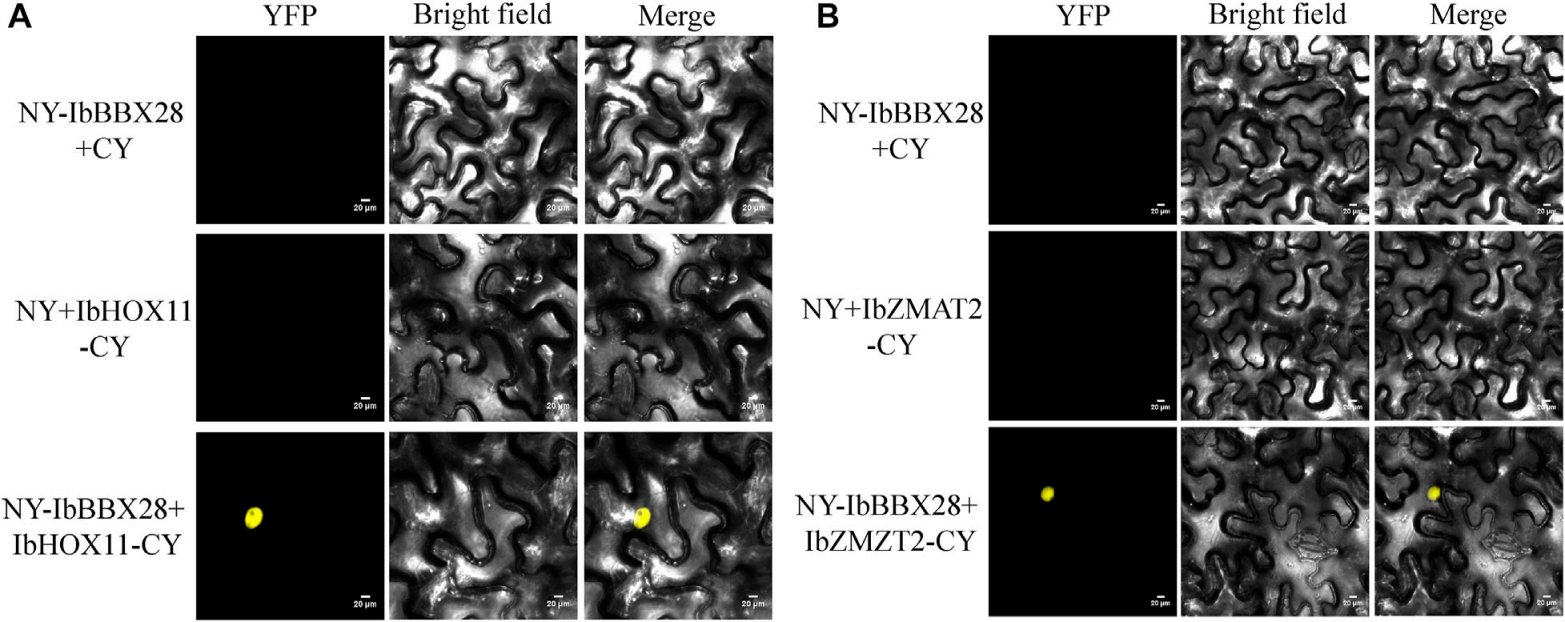
BiFC validation of the IbBBX28 interacting protein. **(A)** BiFC assay of IbBBX28 with IbHOX11. **(B)** BiFC assay of IbBBX28 with IbZMAT2.

In order to study the mechanism of *IbBBX28* involved in stress regulation, the interacting proteins of IbBBX28 were screened in the yeast cDNA library of sweet potato tuberous roots using yeast two-hybrid method. A total of 25 proteins interacting with IbBBX28 were screened (Supplementary Table S5). According to the annotation information of each protein, the proteins of which are of significance and without frame-shifting were selected for interaction verification. Among the interaction proteins, the homeobox-leucine zipper protein HOX11 (IbHOX11) may function in stress response, and studies about zinc finger matrin-type protein 2 (IbZMAT2) in plants were few. Therefore, IbHOX11 and IbZMAT2 were selected for the next validation test. The results showed that pGBKT7-IbBBX28 + pGADT7-IbHOX11 and pGBKT7-IbBBX28 + pGADT7-IbZMAT2 grew well on SD/-Trp/-His/-Leu/-Ade/X-α-Gal deficient medium. This indicates that IbBBX28 interacts with IbHOX11 and IbZMAT2 in the yeast system (Figure 7). To further verify the interaction of IbBBX28 with IbHOX11 and IbZMAT2, a BiFC assay was performed in tobacco leaves (Figure 8). The results showed that no YFP fluorescence signal was detected in the negative controls (NY-IbBBX28 + CY、NY + IbHOX11-CY, and NY + IbZMAT2-CY) after injection into tobacco leaves. However, the YFP fluorescence signal was observed after NY-IbBBX28 + IbHOX11-CY and NY-IbBBX28 + IbZMAT2-CY were injected into tobacco leaves, respectively. This indicated that IbBBX28 interacts with IbHOX11 and IbZMAT2.

## Discussion

B-box transcription factors contain characteristic B-box domains and play an important role in plant growth, development, and stress response (Gangappa and Botto, 2014). The conserved zinc finger structure of the B-box domain can bind to zinc ions to enhance protein stability and play a role in protein and protein interaction (Wang et al., 2021). Many studies have reported that BBX proteins are involved in plant signal transduction pathways and respond to various abiotic stresses, such as low temperature, high salinity, drought, and heat stress (Nagaoka and Takano, 2003; Wang et al., 2013; Yang et al., 2014). *AtBBX28*, a repressor of light signaling, delayed the flowering time in Arabidopsis under long day conditions (Lin et al., 2018; Liu et al., 2020). Furthermore, heterologous expression of strawberry *FaBBX28C1* have been found to negatively regulate the flowering time in Arabidopsis (Ye et al., 2021). In this study, there existed drought response and hormone response *cis*-elements in *IbBBX28*-Pro, including MBS, P-box, ABRE, CGTCA-motif. And the expression of *IbBBX28* in the roots, stems, and leaves of sweet potato was significantly induced under drought stress, suggesting that *IbBBX28* response to drought stress.

To resist environmental stress, plants have evolved a series of regulatory pathways that allow them to respond and adapt the environment. Under adverse conditions, a large amount of ABA is accumulated in plants to enhance stress resistance (Ikegami et al., 2009). The ROS scavenging system can remove the toxic effects caused by ROS and enhance the activity of protective enzymes (Gill and Tuteja, 2010), and improving the ROS scavenging activity can also increase the stress tolerance of plants (Liu et al., 2014; Kikuchi et al., 2015). The protective enzymes of ROS scavenging in plants include CAT, SOD, POD, APX and so on. When under adversity, MDA content increases with the excessive accumulation of superoxide free radicals and hydrogen peroxide. The higher content of MDA can induce cell membrane damage, and the tolerance to stress is reduced in plants (Bao et al., 2009; Kumar et al., 2010). In this study, we found that the activities of the protective enzymes (CAT, SOD, and POD) in the transgenic Arabidopsis were lower than those in the WT, while the content of MDA was higher than that in the WT. The protective enzyme activity and MDA content in the Arabidopsis mutant line *bbx28* were opposite. Moreover, the ROS scavenging enzyme related genes (*AtAPX1*, *AtSOD*, and *AtPOD*) and ABA synthesis-related genes (*AtABA3*, *AtSDR*, and *AtNCED3*) in the transgenic Arabidopsis were lower than those in the WT, while the expression level of these genes in the Arabidopsis mutant line *bbx28* was higher than the WT. Therefore, we predicted that *IbBBX28* negatively regulates drought tolerance in *Arabidopsis thaliana*.

To further explore the mechanism of *IbBBX28* in regulating the stress response in sweet potato, the interacting proteins of IbBBX28 were screened. We found that IbBBX28 interacted with IbHOX11 and IbZMAT2 (Figure 7 and Figure 8). The HOX gene family is involved in several biological processes in plants, such as embryo morphology, meristem development of roots, shoots, and flowers, vascular development, and multiple stress responses, and they are key regulators of plant morphogenesis (Kikuchi et al., 2015). Lui et al. performed transcriptome sequencing of *L. multiflorum* under drought; they found that HOX22 and HOX24 presented high expression levels in leaf samples under drought stress, and they were identified as the enrichment of DEGs in the Intrinsic/Integral component of the Golgi membrane by GO analysis (Liu Q. et al., 2022). These results indicated that IbBBX28 may be involved in drought tolerance by interacting with IbHOX11. ZMAT2 is a matrix protein with zinc finger structure, which plays an important role in keratinocyte differentiation and RNA splicing. Studies on ZMAT mainly focus on human diseases, while there are few related studies in plants (Baral and Rotwein, 2020; Suzuki et al., 2020). The function of IbZMAT2 in the drought response of sweet potato is needs to be clarified in future studies.

The model plant Arabidopsis was often used to study gene function for its short growth cycle and mature transformation system. However, the difference in the genetic background may bring different results. The results of the heterologous gene expression in Arabidopsis may be different from those of the transformation in the background plant, which may interfere with the results. For example, the overexpression of *CmBBX22* delays leaf senescence and improves drought tolerance in Arabidopsis (Liu et al., 2019). However, *CmBBX22* showed reduced drought resistance in transgenic chrysanthemum, which was contrary to the results in Arabidopsis, indicating that *CmBBX22* responded to stress differently in Arabidopsis and *Chrysanthemum* (Liu Y. et al., 2022). Therefore, based on the results in Arabidopsis, we will conduct subsequent experiments in sweet potato to further study the mechanism of *IbBBX28* in response to drought stress.

## Conclusion

In this study, the expression of the *IbBBX28* was induced under drought stress in sweet potato. And *IbBBX28* negatively regulated the drought tolerance in Arabidopsis. Yeast two-hybrid and BiFC verified that IbBBX28 interacted with IbHOX11 and IbZMAT2. IbBBX28 may be involved in regulating the stress response in sweet potato by interacting with IbHOX11 and IbZMAT2.

## Data Availability

The datasets presented in this study can be found in online repositories. The names of the repository/repositories and accession number(s) can be found in the article/Supplementary Material.

## Supplementary material

Supplementary Figure S1 Phylogenetic analysis of *IbBBX28* in different species.
