# Supplementary figures and images for: The sweet potato B-box transcription factor gene IbBBX28 negatively regulates drought tolerance in transgenic Arabidopsis
Source: Front Genet. 2022 Nov 29;13:1077958. doi: 10.3389/fgene.2022.1077958 (PMC9744756; doi:10.3389/fgene.2022.1077958)

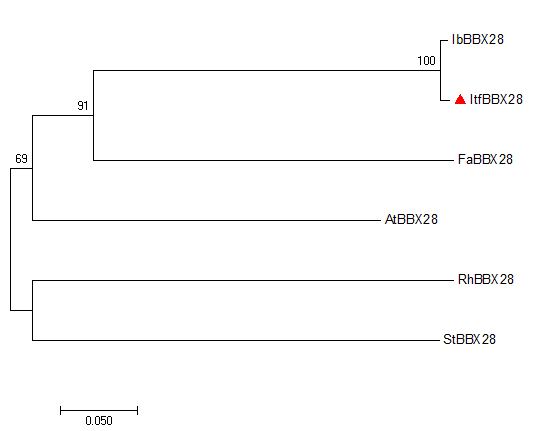

Supplement: Supplementary file 1 [file Image1.tif]
